# S07-1: Why are specific parts of an urban green area used more than others among older adults? A case study utilizing GPS and environmental geographic data

**DOI:** 10.1093/eurpub/ckae114.227

**Published:** 2024-09-26

**Authors:** Kirsi E Keskinen, Eun-Kyeong Kim, Sari Stenholm, Taina Rantanen

**Affiliations:** Faculty of Sport and Health Sciences and Gerontology Research Center, University of Jyväskylä, Jyväskylä, Finland; JYU.Well, University of Jyväskylä, Jyväskylä, Finland; School of Resource Wisdom, University of Jyväskylä, Jyväskylä, Finland; Department of Urban Development and Mobility, Luxembourg Institute of Socio-Economic Research, Esch/Alzette, Luxembourg; Department of Public Health, University of Turku and Turku University Hospital, Turku, Finland; Centre for Population Health Research, University of Turku and Turku University Hospital, Turku, Finland; Faculty of Sport and Health Sciences and Gerontology Research Center, University of Jyväskylä, Jyväskylä, Finland

## Abstract

**Purpose:**

Green areas are known as places attracting physical activity, but less is known about factors contributing to the usage of their specific parts among older adults. Using as study area the ‘Green loop’, an urban green area surrounding Jyväskylä city center, we investigated differences in land use types and on-foot accessibility between ‘Green loop’ parts with different usage intensities among older adults.

**Methods:**

GPS measurements (SenseDocTM 2.0) were conducted over 1-5 days for 231 community-dwelling people over 78 years who lived in Jyväskylä, Finland and participated in the AGNES follow-up study in 2021-22. We identified GPS points indicating active transport and locating in the ‘Green loop’. We generated a point-density map and classified it into three tiers of usage intensity (low/moderate/high). Digiroad data was used to locate walkway entry points and Corine Land Cover data to define land use types (residential/service/industry and transport/nature and green) in the ‘Green loop’. We examined entry point density and, by using the Chi-square test, differences in land use type distributions between the ‘Green loop’ parts with different usage intensities.

**Results:**

More than half (60%) of the ‘Green loop’ area was classified as low-use and only 7 % as high-use. Overall, residential land use type was more common in high than low and moderate-use areas, service more common in low than moderate-use areas, industry and transport more common in moderate than high and low-use areas and more common in high than low-use areas, and green and nature more common in low than high and moderate-use areas (p<.05 for all). Around entry points, service was more common in low than moderate (p<.05) and high-use (p = 0.057) areas. Entry point density was the highest in high and lowest in low-use areas.

**Conclusions:**

Only a limited portion of the ‘Green loop’ area was actively utilized by older adults. Having green spaces located close to residential buildings and more accessible entry points to the green spaces may encourage older adults to spend more time in urban green areas.

**Funding Source:**

European Research Council (TR), Academy of Finland (TR), Ministry of Education and Culture, Finland (TR), Juho Vainio Foundation (KK).

